# Effect of Long-Term Fertilization on Ammonia-Oxidizing Microorganisms and Nitrification in Brown Soil of Northeast China

**DOI:** 10.3389/fmicb.2020.622454

**Published:** 2021-02-04

**Authors:** Fangfang Cai, Peiyu Luo, Jinfeng Yang, Muhammad Irfan, Shiyu Zhang, Ning An, Jian Dai, Xiaori Han

**Affiliations:** ^1^College of Land and Environment, Shenyang Agricultural University, Shenyang, China; ^2^National Engineering Laboratory for Efficient Utilization of Soil and Fertilizer Resources, Tai’an, China; ^3^Northeast Scientific Observation Station of Corn Nutrition and Fertilization of Ministry of Agriculture, Shenyang, China; ^4^Department of Biotechnology, University of Sargodha, Sargodha, Pakistan

**Keywords:** ammonia-oxidizing archaea, ammonia-oxidizing bacteria, potential nitrification rate, brown soil, long-term fertilization

## Abstract

The objective of this study was to find out changes in ammonia oxidation microorganisms with respect to fertilizer as investigated by real-time polymerase chain reaction and high-throughput sequencing. The treatments included control (CK); chemical fertilizer nitrogen low (N) and high (N_2_); nitrogen and phosphorus (NP); nitrogen phosphorus and potassium (NPK) and organic manure fertilizer (M); MN; MN_2_; MNPK. The results showed that long-term fertilization influenced soil fertility and affected the abundance and community of ammonia-oxidizing microorganisms by changing the physical and chemical properties of the soil. The abundance and community structure of ammonia-oxidizing archaea (AOA) and ammonia-oxidizing bacteria (AOB) was influenced by soil organic carbon, total nitrogen, total soil phosphorus, available phosphorus, available potassium, and soil nitrate. Soil environmental factors affected the nitrification potential by affecting the structure of ammonia-oxidizing microorganisms; specific and rare AOA and AOB rather than the whole AOA or AOB community played dominant role in nitrification.

## Introduction

Agricultural nitrogen cycling is a crucial part in global nitrogen cycling, and ammoxidation is one of critical process of agricultural nitrogen cycling, which turns ammonia to nitrite and then to nitrate. Ammonia-oxidizing organisms, limiting the rate, play an important role in the first step of nitrification. As Könneke et al. (2005) found that archaea could oxidize ammonia to nitrite, researchers studied the comparison between ammonia-oxidizing archaea (AOA) and ammonia-oxidizing bacteria (AOB) about which one was more important under different environmental conditions (Adair and Schwartz, 2008; Chen et al., 2008, 2015; Li et al., 2010; Tao et al., 2017). However, it is controversial whether AOA or AOB play a dominant role under different environmental conditions (Li et al., 2010; Liu et al., 2015; Gan et al., 2016), which may be due to that ammonia-oxidizing microorganisms community structure were affected by physiochemical properties of soil (Li et al., 2010; Huang et al., 2013). In a previous study, AOA might be dominant within nitrogen cycling under extreme environmental conditions (Erguder et al., 2009); AOA rather than AOB could adapt to oxygenated/hypoxic water conditions (Liu et al., 2015). However, AOB rather than AOA dominated in potential nitrification in other environment (Dang et al., 2018). Different NH_4_^+^-N concentration and different levels of total carbon and total nitrogen in soils influenced the composition and AOA and AOB abundance in various environmental conditions (Huang et al., 2013; Li and Gu, 2013; Wang et al., 2013).

Long-term fertilization, a good way to investigate agricultural soil, changed physicochemical properties and microbial community structure, such as soil pH, dissolved organic carbon (DOC), available phosphorus, etc. (Luo et al., 2015). Crop rotation could also change the physiochemical properties of soil and microbial community structure (Belay et al., 2002; Bhattacharyya et al., 2010). Whether fertilization changed the ammonia-oxidizing microorganisms’ community structure and quantities, which ammonia-oxidizing microorganisms play a dominant role in the nitrification process under the condition of long-term rotation and fertilization is still unclear. In this study, we evaluated the effects of fertilization on the changing of AOA and AOB because of a long-term (38 years) rotation and fertilization experiment. Our objectives were (i) to determine whether AOA and AOB had different responses to variations under the condition of long-term fertilization and rotation, (ii) to identify which taxa play a more crucial role during nitrification process, and (iii) to assess the relative contribution of those variables to changes in AOA and AOB activities and function.

## Materials and Methods

### Experimental Site and Soil Sampling

This 38 years fertilization experiment was established in 1979; the field soil properties and block design were described by Hua et al. (2020). The field experiment was conducted under a rotation of maize–maize–soybean. The crop-growing season started at the end of April and ended at the end of September. In this study, we have chosen nine treatments as follows: CK (no fertilizer), N (mineral nitrogen fertilizer), N_2_ (high mineral nitrogen fertilizer), NP (N and phosphate fertilizer), NPK (N, P, and potassium fertilizer), and M (manure), MN, MN_2_, MNPK. N was applied in the form of urea, whereas P was calcium superphosphate, and K was potassium sulfate, and the manure fertilizer used was pig manure; the specific application rates are detailed in Supplementary Table S1. Soil samples were collected from surface soil layer (0–20 cm) and plow pan soil layer (20–40 cm) on each plot on October 7, 2016. Three soil cores (5 cm diameter) were taken from each layer of soil in each plot, respectively. All samples were shifted through a 2.0 mm sieve. Some part of fresh soil samples was used for DNA extraction, whereas some part was used to determine the soil moisture content, and the rest was air-dried and ground for physiochemical properties determination.

### Chemical Analysis

Soil properties determined was soil pH, DOC, soil organic carbon (SOC), total nitrogen (TN), total phosphorus (TP), total potassium (TK), and available phosphorus (AP) and potassium (AK), as described earlier (Luo et al., 2015). The fresh soil samples were used to determine soil ammonium (NH_4_^+^-N) and nitrate (NO_3_^–^-N), extracted with 1 M KCl and determined by a continuous flow analyzer (AutoAnalyzer 3; SEAL Analytical Ltd., Germany). Soil potential nitrification rate (PNR) was determined using the method of Kandeler (1995).

### DNA Extraction and Quantitative Polymerase Chain Reaction Amplification

DNA was extracted from soil (0.250 g) using the PowerSoil^TM^ DNA Isolation Kit (MoBio Laboratories Inc., United States). Purified DNA extracts were stored at −20°C until further determination. Amplification of archaeal and bacterial *amo*A genes was performed using the primer, which is listed in Supplementary Table S2. The reaction mixture (20 μL) contained 1 μL of DNA template (20–40 ng per reaction), 0.8 μL of forward and reverse primers (10 μM), respectively, and 10 μL of TB Green Premix Ex Taq (2X) 10 μL (Takara Biomedical Technology Co. Ltd., Beijing, China). The quantitative polymerase chain reaction (qPCR) amplification procedure of *amo*A gene was performed as prime denaturation at 95°C for 30 s, followed with 40 cycles of denaturation for 5 s at 95°C, annealing for 30 s at 55°C, and extension for 30 s at 72°C. The qPCR amplification procedure of arch-amoA gene was performed as prime denaturation at 94°C for 30 s, followed with 45 cycles of denaturation for 30 s at 94°C, annealing for 60 s at 55°C, and extension for 60 s at 72°C.

### High-Throughput Sequencing

The pure PCR product equimolar and paired end was sequenced according to the standard protocols by Majorbio Bio-Pharm Technology Co., Ltd. (Shanghai, China) (Zhang et al., 2020). The raw *amo*A gene sequencing reads were demultiplexed, quality-filtered by fast p version 0.20.0 (Chen et al., 2018), and merged by FLASH version 1.2.7 (Magoč and Salzberg, 2011) with the same following criteria as described by Zhang et al. (2020).

UPARSE version 7.1 clustering was used to identify and remove chimeric sequences of operation classification unit (OTUs) with 97% similarity truncation (Edgar, 2013). RDP version 2.2 was used for classification analysis of *amo*A database, and the confidence threshold was 0.7 (Wang et al., 2007).

### Statistical Analysis

Redundancy analysis (RDA) (CANOCO 4.5 for Windows) was used to evaluate the relationships among high-throughput sequencing results, quantity PCR results, and physicochemical properties of fertilization treatments. The measured data were used to analyze the variation, using IBM SPSS Statistics 19.0 for Windows (IBM, Inc., Armonk, NY, United States). Analysis of variance and non-metric multidimensional scaling (NMDS) analysis (calculated by Qiime, and analysis and mapping by Vegan software package for R) were used to analyze the environmental factors and microbes index. A probability level of 5% was adopted for accepting or rejecting null hypotheses.

## Results

### Soil Physicochemical Properties

Long-term fertilization prominently changed the brown soil properties from both physical and chemical aspects (Table 1). Soil pH was varied from 5.03 to 6.95 and 5.78 to 6.86; SOC contents varied from 13.91 to 23.32 g ⋅ kg^–1^ and 10.42 to 20.51 g ⋅ kg^–1^; AP contents varied from 1.65 to 136.75 mg ⋅ kg^–1^ and 1.45 to 117.90 mg ⋅ kg^–1^; and NO_3_^–^-N contents varied from 2.65 to 27.32 mg ⋅ kg^–1^ and 1.50 to 19.80 mg ⋅ kg^–1^. PNR was varied from 0.86 to 5.06 mg ⋅ kg^–1^ ⋅ d^–1^ and 0.93 to 3.99 mg ⋅ kg^–1^ ⋅ d^–1^; DOC contents varied from 21.83 to 36.04 mg ⋅ kg^–1^ and 3.72 to 14.05 mg ⋅ kg^–1^ in surface arable soil layer (0–20 cm) and plow pan soil layer (20–40 cm), respectively. The application of manure significantly (*P* < 0.05) increased the soil pH, PNR, and the stocks of SOC, TN, TP, AP, AK, and NO_3_^–^-N of soil in both surface arable soil layer and plow pan soil layer. It had no remarkable difference (*P* > 0.05) in the stocks of TK and NH_4_^+^-N in all fertilization treatments.

**TABLE 1:** Physicochemical properties of soils in different fertilization treatments after 38 years of long-term fertilization.

	Soil layers	CK	N	N_2_	NP	NPK	M	MN	MN_2_	MNPK
pH	0–20 cm	5.97 ± 0.1^c^	5.24 ± 0.07^e^	5.03 ± 0.07^f^	5.72 ± 0.12^d^	5.77 ± 0.08^d^	6.95 ± 0.07^a^	6.2 ± 0.02^b^	5.93 ± 0.07^c^	6.13 ± 0.02^b^
	20–40 cm	5.93 ± 0.01^e^	6.06 ± 0.04^d^	5.78 ± 0.06^f^	6.42 ± 0.07^c^	6.14 ± 0.07^d^	6.86 ± 0.03^a^	6.56 ± 0.03^b^	6.55 ± 0.07^b^	6.42 ± 0.05^c^
TN (g•kg^–1^)	0–20 cm	0.99 ± 0.01^e^	1.08 ± 0.03^d^	0.99 ± 0.04^e^	1.15 ± 0.07^c^	1.07 ± 0.01^d^	1.53 ± 0.01^a^	1.46 ± 0.02^b^	1.56 ± 0.02^a^	1.55 ± 0.02^a^
	20–40 cm	0.87 ± 0.01*de*	0.73 ± 0.02^f^	0.84 ± 0.02*ef*	0.82 ± 0.05^g^	0.83 ± 0.02*ef*	0.92 ± 0.01^c^	0.88 ± 0.01^d^	0.98 ± 0.03*bc*	1.17 ± 0.01^a^
SOC (gkg•^–1^)	0–20 cm	13.91 ± 0.03^f^	15.26 ± 0.03^d^	14.44 ± 0.51^e^	17.29 ± 0.68^c^	16.33 ± 0.07^c^	23.31 ± 0.17^a^	21.6 ± 0.02^b^	23.32 ± 0.11^a^	22.82 ± 0.01^a^
	20–40 cm	12.15 ± 0.03^f^	10.42 ± 0.18^h^	11.59 ± 0.12^g^	12.61 ± 0.01^e^	12.49 ± 0.13^e^	14.5 ± 0^c^	13.34 ± 0.14^d^	15.35 ± 0.04^b^	20.51 ± 0.23^a^
TP (g•kg^–1^)	0–20 cm	0.2 ± 0.01^e^	0.22 ± 0.01^e^	0.21 ± 0.03^e^	0.27 ± 0.02^d^	0.27 ± 0.03^d^	0.7 ± 0.04^b^	0.6 ± 0.01^c^	0.68 ± 0.03^b^	0.83 ± 0.02^a^
	20–40 cm	0.24 ± 0.02^f^	0.24 ± 0.01^f^	0.23 ± 0.01^f^	0.26 ± 0.01^f^	0.31 ± 0.01^e^	0.48 ± 0.03^b^	0.35 ± 0^d^	0.43 ± 0^c^	0.63 ± 0.02^a^
TK (g•kg^–1^)	0–20 cm	22.11 ± 2.42*abcde*	20.26 ± 1.03^e^	21.51 ± 2.29*bcde*	20.81 ± 0.77*de*	21.06 ± 1.3*cde*	23.38 ± 0.4*ab*	24.17 ± 1.02^a^	23.8 ± 0.38*ab*	23.27 ± 0.51*abcd*
	20–40 cm	23.85 ± 0.51*ab*	22.29 ± 1.64^b^	23.08 ± 0.59*ab*	24.11 ± 0.56*ab*	23.17 ± 0.84*ab*	24.31 ± 0.28^a^	23.68 ± 1.04*ab*	23.21 ± 1.17*ab*	23.7 ± 1.26*ab*
AP (mg•kg^–1^)	0–20 cm	4.05 ± 0.2^f^	1.65 ± 0.69^f^	2.74 ± 0.4^f^	10.89 ± 0.89*ef*	18.78 ± 3.68^e^	98.12 ± 9.55^c^	74.21 ± 15.44^d^	100.63 ± 1.87^b^	136.75 ± 0.82^a^
	20–40 cm	3.32 ± 0.31^e^	1.62 ± 0.27^e^	1.45 ± 0.15^e^	5.46 ± 0.82^e^	4.57 ± 0.27^e^	68.01 ± 5.35^b^	31.61 ± 0.97^d^	42.58 ± 2.12^c^	117.9 ± 6.69^a^
AK (mg•kg^–1^)	0–20 cm	86.83 ± 6.45^e^	79.21 ± 0.95^e^	58.58 ± 2.76^f^	89.73 ± 11.9^e^	148.38 ± 9.19^c^	114.01 ± 13^d^	153.59 ± 7.16*bc*	164.91 ± 10.62*ab*	169.5 ± 9.23^a^
	20–40 cm	73.25 ± 4.63*cd*	73.58 ± 4.01*cd*	72.91 ± 4.94^d^	62.23 ± 3.79^e^	77.25 ± 8.1*cd*	87.6 ± 1^b^	77.25 ± 1.53*cd*	83.6 ± 5.58*bc*	104.63 ± 9.86^a^
NH_4_^+^-N (mg•kg^–1^)	0–20 cm	3.62 ± 0.2*ab*	3.49 ± 0.2^b^	3.45 ± 0.08^b^	4.07 ± 0.55^a^	3.32 ± 0.13^b^	3.4 ± 0.17^b^	3.71 ± 0.33*ab*	4.08 ± 0.37^a^	3.37 ± 0.08^b^
	20–40 cm	3.54 ± 0.2^a^	3.62 ± 0.27^a^	3.47 ± 0.04^a^	3.59 ± 0.27^a^	3.67 ± 0.37^a^	3.34 ± 0.03^a^	3.64 ± 0.12^a^	3.58 ± 0.19^a^	3.5 ± 0.06^a^
PNR (mg•kg^–1^•d^–1^)	0–20 cm	0.86 ± 0.02^h^	1.83 ± 0.07^e^	1.4 ± 0.08^g^	1.6 ± 0.05^f^	1.68 ± 0*ef*	5.06 ± 0.17^a^	2.59 ± 0.05^c^	2.4 ± 0.06^d^	3.46 ± 0.11^b^
	20–40 cm	1.16 ± 0.04^d^	0.96 ± 0.04^e^	1.45 ± 0.11^c^	0.54 ± 0.03^f^	0.64 ± 0.1^f^	3.99 ± 0.19^a^	2.54 ± 0.04^b^	1.61 ± 0.11^c^	2.52 ± 0.09^b^
NO_3_^–^-N (mg•kg^–1^)	0–20 cm	2.56 ± 0.15^c^	8.12 ± 0.49^b^	7.00 ± 0.31*bc*	5.65 ± 0.49*bc*	5.01 ± 0.3*bc*	27.32 ± 2.15^a^	23.22 ± 6.44^a^	22.45 ± 1.72^a^	25.51 ± 3.79^a^
	20–40 cm	1.5 ± 0.31^f^	5.23 ± 0.34^e^	7.76 ± 0.56^d^	2.17 ± 0.75^f^	2.49 ± 0.31^f^	19.8 ± 0.8^a^	12.88 ± 1.19^c^	16.76 ± 1.71^b^	12.53 ± 1.21^c^
DOC (mg•kg^–1^)	0–20 cm	15.58 ± 2.9^e^	25.56 ± 2.02*cd*	36.04 ± 3.48^a^	34.59 ± 5.85*ab*	26.55 ± 1.78*cd*	22.26 ± 4.26^d^	21.83 ± 0.27^d^	29.03 ± 0.9*bc*	27.27 ± 5.44*cd*
	20–40 cm	3.72 ± 0.04^c^	12.44 ± 1.12^a^	4.17 ± 1.38^c^	4.66 ± 2.35^c^	5.19 ± 0.82^c^	4.88 ± 1.22^c^	9.21 ± 2.53^b^	14.05 ± 2.51^a^	6.3 ± 1.39^c^
SMC (%)	0–20 cm	20.76 ± 0.1^c^	20.99 ± 0.2^c^	20.30 ± 0.2^c^	18.16 ± 1.1^d^	19.94 ± 0.7^c^	23.72 ± 1.0^a^	22.54 ± 0.2^b^	22.36 ± 0.1^b^	22.68 ± 0.4^b^
	20–40 cm	21.98 ± 0.5*abc*	21.33 ± 1.1*abc*	20.68 ± 0.4^c^	21.42 ± 1.4*abc*	21.23 ± 0.7*bc*	21.93 ± 0.7*abc*	21.50 ± 0.8*abc*	22.66 ± 0.4*ab*	22.79 ± 0.4^a^

*CK, no fertilizer, N, mineral nitrogen fertilizer; N_2_, high mineral nitrogen fertilizer; NP, mineral nitrogen and phosphate fertilizer; NPK, mineral nitrogen, phosphate and potassium fertilizer; M, pig manure; MN, pig manure and mineral nitrogen fertilizer; MN_2_, pig manure and high mineral nitrogen fertilizer; MNPK, pig manure, mineral phosphate, potassium fertilizer and mineral nitrogen fertilizer; SOC, soil organic carbon; TN, total nitrogen; TP, total phosphorus; TK, total potassium; AP, available phosphorus; AK, available potassium; NH_4_^+^-N, ammonia nitrogen; NO_3_^–^-N, nitrate nitrogen; PNR, potential nitrite rate; DOC, dissolved organic carbon; SMC, soil moisture carbon. The mean value ± SD (n = 3). Different letters in the same row represent significantly differences among fertilization treatments (LSD method, and p < 0.05).*

In addition to organic fertilizer treatments, contents of SOC in chemical phosphorus fertilizer treatments (NP, NPK) were significantly higher (*P* < 0.05) than that in N and N_2_ treatments. The content of PNR in organic fertilizer treatments was significantly higher (*P* < 0.05) than that in chemical fertilizer treatments, and that in surface arable soil was significantly higher (*P* < 0.05) than that in plow pan soil. It was highest in M treatment in both soil layers. The stock of DOC in surface arable soil in the M treatment was the highest in all treatments, and contents of DOC in plow pan soil in N, MN, and MN_2_ treatments were the highest in all treatments.

### Abundance and Community Structures of AOB and AOA in Soil

**FIGURE 1:**
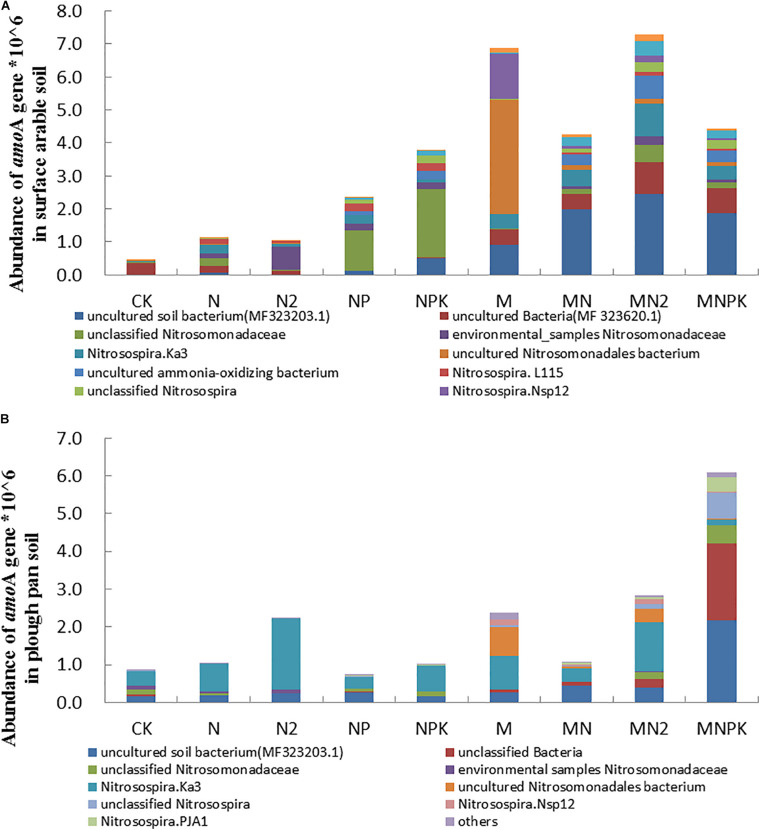
The abundance and community structure in species level of ammonia-oxidizing bacteria in different treatments in surface arable soil **(A)** and plow pan soil **(B)**, respectively. The mean value ± *SD* (LSD method, and *p* < 0.05, *n* = 3).

The *aom*A gene (AOB) quantified by qPCR ranging from 4.68 × 10^5^ to 7.28 × 10^6^ copy numbers per gram of dry soil in surface arable soil layer (Figure 1A) and 7.44 × 10^5^ to 6.1 × 10^6^ copy numbers per gram of dry soil in plow pan soil layer (Figure 1B) in this experiment. The abundance of *amo*A gene in both soil layers in organic fertilizer treatments was prominently higher (*P* < 0.05) than that in chemical fertilizer treatments. Except for N_2_ and MNPK treatments, the abundance of *amo*A gene in surface arable soil layer was higher than that in plow pan soil layer.

Community structures of AOB were similar in both of two soil layers under long-term fertilization (Figures 1A,B). The abundance of uncultured bacterium MF323203.1 (MNPK 23.49 and 50.90%, MN 24.99 and 10.21%, MN_2_ 30.86 and 8.91%, M 11.52 and 5.92% in surface arable soil layer and plow pan soil layer, respectively), uncultured bacterium MF323620.1 (MNPK 22.25 and 81.09%, MN 14.22 and 3.88%, MN_2_ 28.83 and 9.14%, M 13.95 and 3.40% in surface arable soil layer and plow pan soil layer, respectively), and *Nitrosospira* PJA1 (MNPK 19.00 and 74.35%, MN 22.97 and 9.78%, MN_2_ 37.87 and 10.68%, M 3.27 and 0.76% in surface arable soil layer and plow pan soil layer, respectively) in organic fertilizer treatments was prominently higher (*P* < 0.05) than that in other treatments. The abundance of unclassified *Nitrosomonadaceae* in NP (27.70 and 7.67% in surface arable soil layer and plow pan soil layer, respectively), NPK (46.70 and 10.24% in surface arable soil layer and plow pan soil layer, respectively), and *Nitrosospira*.L115 in NP (27.34% in surface arable soil layer) and NPK (25.24% in surface arable soil layer) treatments were significantly higher (*P* < 0.05) than that in other treatments. The abundance of *Nitrosomonadaceae* (40.78 and 40.02% in surface arable soil layer and plow pan soil layer, respectively) was the highest in N_2_ treatment. The abundance of uncultured *Nitrosomonadales* bacterium was the highest in M treatment (87.62 and 62.64% in surface arable soil layer and plow pan soil layer, respectively). The abundance of *Nitrosospira*.Ka3, uncultured AOB and *Nitrosospira*.Nsp12 in fertilizer treatments were prominently higher (*P* < 0.05) than that in CK treatment (*Nitrosospira*.Ka3 0.62 and 5.82%, uncultured AOB 0.73 and 0.07% in surface arable soil layer and plow pan soil layer, respectively). Unclassified *Nitrosospira* in surface arable soil layer was different from that in plow pan soil layer. The quantities of unclassified *Nitrosospira* were dominantly higher in NP (11.52%), NPK (20.01%) treatments, and organic fertilizer treatments (MNPK 23.11%, MN 11.86%, MN2 25.40%, M 3.83%) than that in other treatments in surface arable soil, whereas the quantities of unclassified *Nitrosospira* were significantly higher (*P* < 0.05) in organic fertilizer treatments (MNPK 69.69%, MN 5.09%, MN_2_ 11.79%, M 5.68%) than that in chemical fertilizer treatments in plow pan soil.

**FIGURE 2:**
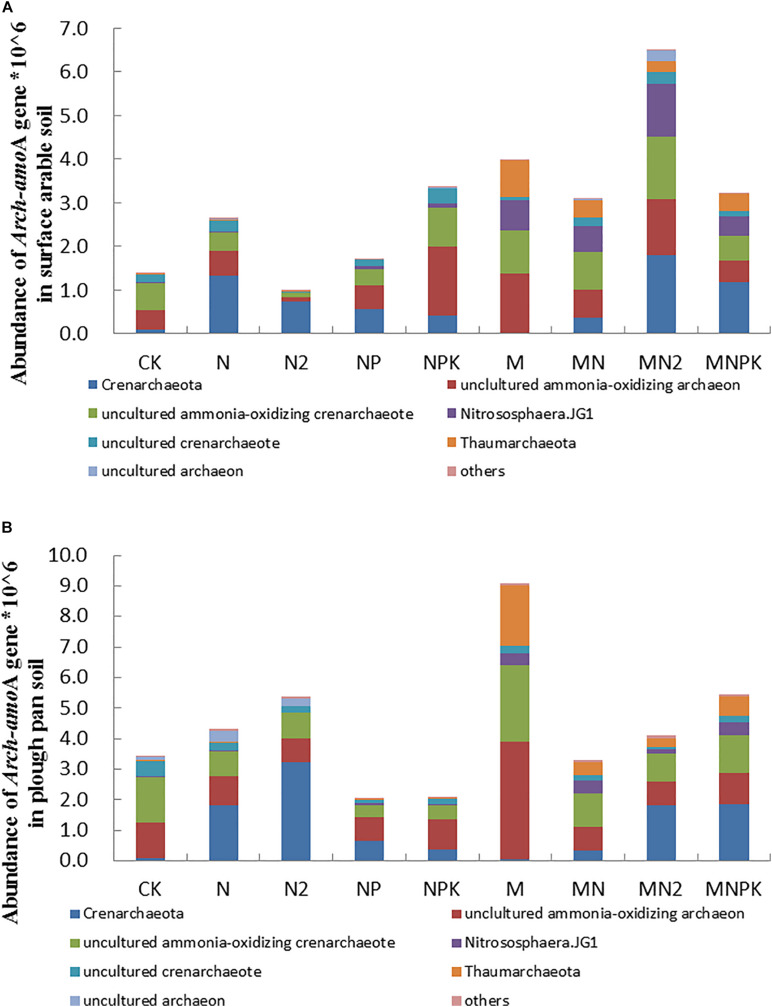
The abundance and community structure in species level of ammonia-oxidizing archaea in different treatments in surface arable soil **(A)** and plow pan soil **(B)**, respectively. The mean value ± *SD* (LSD method, and *p* < 0.05, *n* = 3).

The *arch-amo*A gene (AOA) quantified by qPCR ranging from 9.82 × 10^5^ to 6.49 × 10^6^ copy numbers per gram of dry soil in surface arable soil layer (Figure 2A) and 2.07 × 10^6^–9.09 × 10^6^ copy numbers per gram of dry soil in plow pan soil layer (Figure 2B). The abundance of *arch-amo*A gene of soil in MN_2_ treatment was significantly higher (*P* < 0.05) than that in other treatments in surface arable soil layer, and the abundance of *arch-amo*A gene of soil in M treatment was significantly higher (*P* < 0.05) than that in other treatments in plow pan soil layer. There was no prominent difference (*P* > 0.05) in *arch-amo*A gene abundance in surface arable soil layer of N, NPK, MN, and MNPK treatments. The abundance of *arch-amo*A gene in NP, N_2_, and CK treatments were significantly lower (*P* < 0.05) than that in other treatments in surface arable soil. The abundance of *arch-amo*A gene in M, MNPK, N_2_, and N treatments were significantly higher than CK treatment, and CK treatment had no significant difference with MN and MN_2_ treatments in plow pan soil. Except for MN_2_ and NPK treatments, the abundance of *arch-amo*A gene in surface arable soil layer was significantly higher (*P* < 0.05) than that in plow pan soil layer.

Community structures of AOA were similar in both of two soil layers under long-term fertilization (Figures 2A,B). The abundance of *Crenarchaeota* in M (0.37 and 0.56% in surface arable soil layer and plow pan soil layer, respectively) and CK (1.28 and 0.93% in surface arable soil layer and plow pan soil layer, respectively) treatments was dominantly lower (*P* < 0.05) than that in other treatments; MN_2_ treatment (27.69%) was the highest than other treatments in surface arable soil, and N_2_ treatment (31.58%) was the highest treatment than other treatments in plow pan soil. It indicated that excessive nitrogen was good to *Crenarchaeota*, and the increment of *Crenarchaeota* increased with the increase of chemical nitrogen fertilizer; in opposite, organic manure alone inhibited *Crenarchaeota*. The abundance of uncultured AOA in NPK (22.22 and 9.11% in surface arable soil layer and plow pan soil layer, respectively) and M (19.31 and 34.69% in surface arable soil layer and plow pan soil layer, respectively) treatments was dominantly higher (*P* < 0.05) than that in other treatments. The abundance of *Nitrososphaera*.JG1 (MNPK 13.75 and 27.33%, MN 18.69 and 27.70%, MN_2_ 38.52 and 10.08%, M 22.21 and 25.37% in surface arable soil layer and plow pan soil layer, respectively) and *Thaumarchaeota* (MNPK 20.50 and 17.97%, MN 20.44 and 12.88%, MN_2_ 12.68 and 8.18%, and M 44.44 and 56.92% in surface arable soil layer and plow pan soil layer, respectively) in organic fertilizer treatments was significantly higher (*P* < 0.05) than that in chemical fertilizer treatments. The abundance of uncultured ammonia-oxidizing *Crenarchaeote* and uncultured *Crenarchaeote* were different between two soil layers. The abundance of uncultured ammonia-oxidizing *Crenarchaeote* in organic manure treatments (MNPK 9.41%, MN 13.88%, MN_2_ 22.87%, M 15.64%) was significantly higher (*P* < 0.05) than that in other treatments in surface arable soil, whereas that in M (15.21%) and CK (25.67%) treatments was significantly higher (*P* < 0.05) as compared to other treatments in plow pan soil. The abundance of uncultured *Crenarchaeota* in NPK (19.75%), N (15.89%), MN (12.37%), and MN_2_ (17.13%) was significantly higher (*P* < 0.05) than that in other treatments in surface arable soil, whereas that in CK treatment (25.95%) was significantly higher (*P* < 0.05) with respect to other treatments in plow pan soil.

### Community Structures of AOB and AOA in Soil Under Relative Abundance

The relative abundance of AOB community structures was different between surface arable soil with plow pan soil under long-term fertilization (Supplementary Figures S1A,B). The relative quantity of *Nitrosospira*.Ka3 in CK treatment was prominently lower than that in other treatments in surface arable soil (Supplementary Figure S1A). The relative abundance of unclassified *Nitrosomonadaceae*, unclassified environmental samples *Nitrosomonadaceae*, and *Nitrosospira*.L115 in chemical treatments was prominently higher than that in other treatments, and the relative abundance of unclassified environmental samples *Nitrosomonadaceae* in N and N_2_ treatments was prominently higher as compared to other treatments. The relative abundance of uncultured bacterium MF323203.1 in MNPK, MN, and MN_2_ treatments was significantly higher than that in other treatments. It indicated that application of organic in combination with chemical fertilizer could increase the relative quantity of uncultured AOB. The relative abundance of unclassified *Nitrosomonadaceae*, unclassified environmental samples *Nitrosomonadaceae* in CK treatment, was significantly higher than that in other treatments in plow pan soil (Supplementary Figure S1B). The relative abundance of *Nitrosospira*.Ka3 in chemical fertilizer treatments was significantly higher than that in other treatments. The relative abundance of uncultured *Nitrosomonadales* bacterium and *Nitrosospira*.Nsp12 in M treatment was prominently higher than that in other treatments. The relative abundance of unclassified *Nitrosospira*, *Nitrosospira*.PJA1 in N, N_2_, M, and CK treatments was significantly lower among all other treatments. The relative abundance of AOA community structures was similar in both soil layers under long-term fertilization (Supplementary Figures S2A,B). The relative abundance of *Crenarchaeota* in M and CK treatments was dominantly lower than that in other treatments. *Crenarchaeota* in chemical fertilizer treatments was significantly higher than that in organic manure treatments; N_2_ treatment was higher than N treatment; MN_2_ treatment was higher than MN treatment. The relative abundance of uncultured ammonia-oxidizing *Crenarchaeote* and uncultured *Crenarchaeote* in CK treatment was significantly higher than that in other treatments. Uncultured *Crenarchaeote* of relative abundance in chemical fertilizer treatments was higher than that in organic fertilizer treatments. The relative quantity of *Thaumarchaeota* in M treatment was higher than that in other treatments, whereas the relative abundance of *Nitrososphaera*.JG1 in organic manure treatments was significantly higher than that in chemical fertilizer treatments. It indicated that application of organic manure fertilizer increased the quantity.

### Shannon Index of AOA and AOB

**FIGURE 3:**
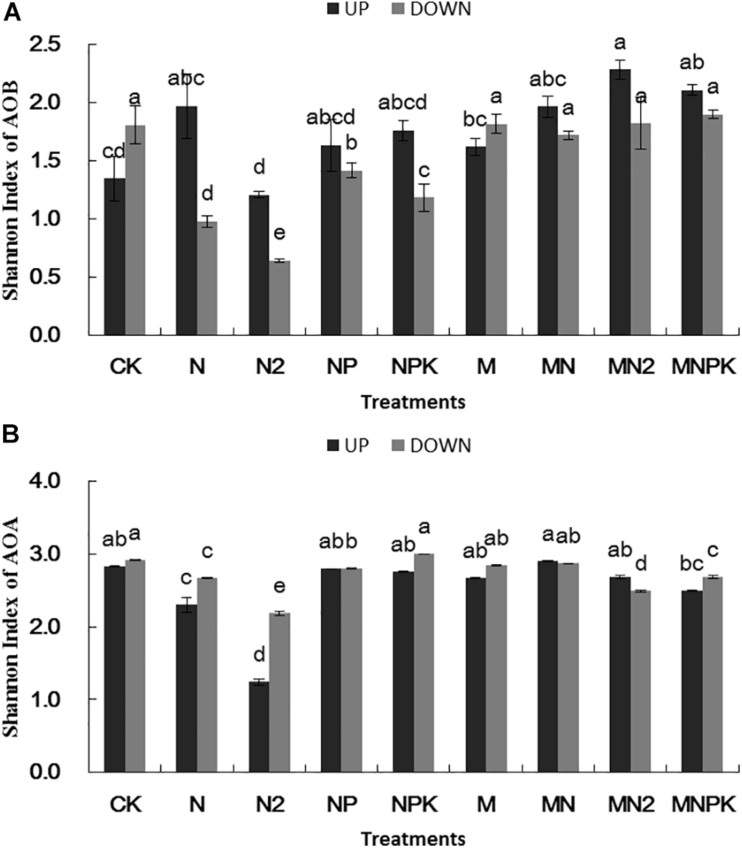
Shannon index of ammonia-oxidizing bacteria **(A)** and archaea **(B)** in different treatments in surface arable soil and plow pan soil, respectively. The mean value ± *SD* (*n* = 3). Different letters represent significant differences among fertilization treatments (LSD method, and *p* < 0.05). UP represented surface arable soil (0–20 cm), and Down represented plow pan soil (20–40 cm).

Shannon index of AOB (Figure 3A) in the N_2_ treatment was significantly lower (*P* < 0.05) than other treatments in both of two soil layers. Shannon index of AOB was higher in surface arable soil than that in plow pan soil. There was no significant difference (*P* > 0.05) between organic fertilizer treatments and CK treatment in Shannon index of AOB in plow pan soil, whereas the Shannon index of AOB was higher in organic manure treatments than that in chemical fertilizer treatments.

Shannon index of AOA (Figure 3B) in N and N_2_ treatments was significantly lower (*P* < 0.05) than that in other treatments in surface arable soil layer, whereas Shannon index of AOA in N, N_2_, and MN_2_ treatments in plow pan soil layer was significantly lower (*P* < 0.05) than that in other treatments. Shannon index of AOA in plow pan soil layer was higher than that in surface arable soil layer.

### NMDS Analysis of AOA and AOB

**FIGURE 4:**
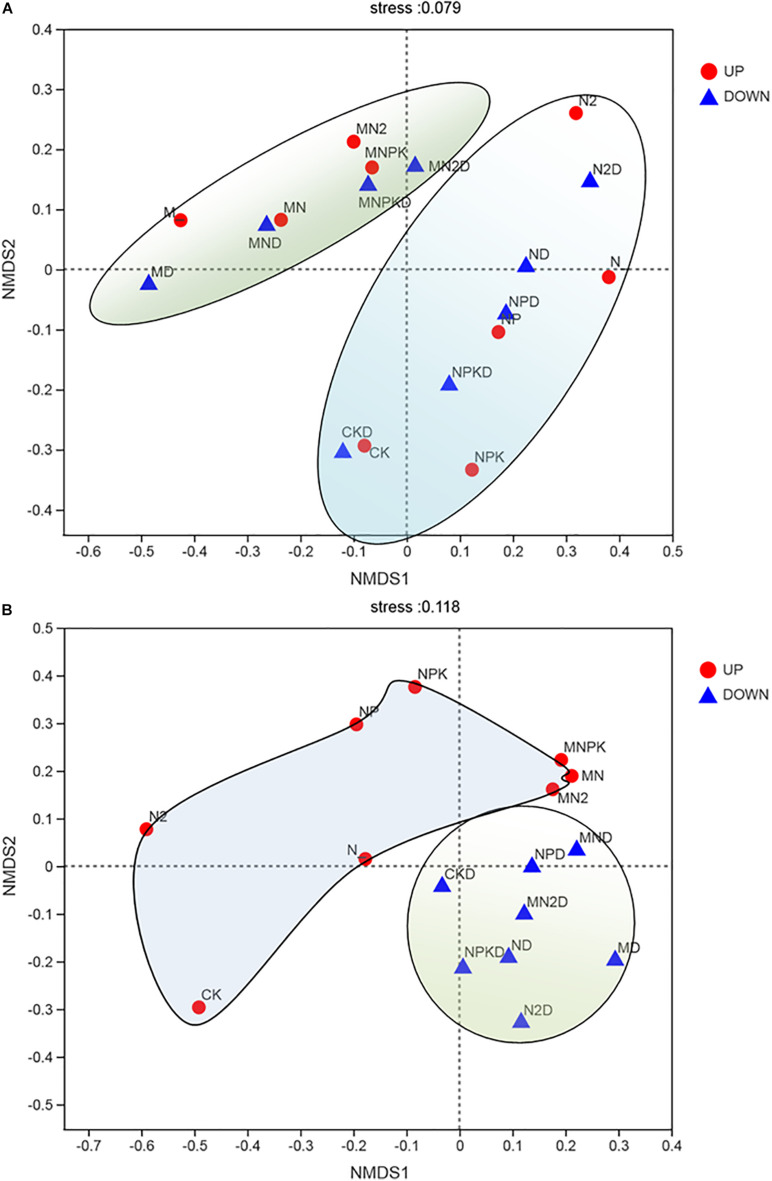
Non-metric multidimensional scaling (NMDS) of AOA **(A)** and AOB **(B)** in two soil layers under different fertilization treatments. UP represented surface arable soil (0–20 cm) and Down represented plow pan soil (20–40 cm). Points of different colors or shapes represent samples of different groups. The closer the two sample points are, the more similar the species composition of the two samples. The abscissa and ordinate indicate the relative distance and have no practical meaning. Stress: Test the pros and cons of NMDS analysis results. It is generally believed that when stress is less than 0.2, it can be represented by a two-dimensional dot diagram of NMDS, which has a certain explanatory meaning; when stress is less than 0.1, it can be considered as a good ranking.

In order to determine whether long-term fertilization and soil layers were related to changes in soil ammonia-oxidizing microbial community structure, we used NMDS based on Bray–Curtis dissimilarities to analyze the overall archaeal and bacterial community structural changes in Figure 4. The NMDS ordinations showed that the AOA (Figure 4A) community structure was divided into two parts: the organic fertilizer treatment and chemical fertilizer treatments. The community structure of AOB (Figure 4B) had a significant correlation with the soil layer, and the community structure of AOB was divided into two parts: the surface arable soil and the plow pan soil.

### The Relationship Among Soil Physicochemical Properties, Abundance, and Community Structure of AOA and AOB

**FIGURE 5:**
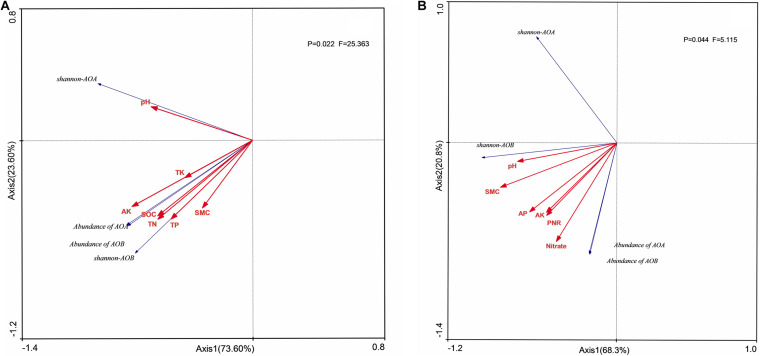
Redundancy analysis (RDA) of AOB and AOA in diversity and quantity in surface arable soil layer **(A)** and plow pan soil layer **(B)**. RDA correlation plot showed changes in ammonia oxidizers diversity and quantity explained by soil environmental factors. SOC, soil organic carbon; TN, total nitrogen; TP, total phosphorus; TK, total potassium; AP, available phosphorus; AK, available potassium; NO_3_^–^-N, nitrate nitrogen; PNR, potential nitrite rate; SMC, soil moisture carbon.

The quantities of AOB in both soil layers (Figure 5) and AOA in surface arable soil layer (Figure 5A) had significant positive correlation (*P* < 0.05) with stocks of SOC, TN, TP, AP, AK, and NO_3_^–^-N under long-term fertilization, and the quantities of AOB in surface soil (Figure 5A) had significant positive correlation (*P* < 0.05) with PNR, whereas the quantity of AOA had significant positive correlation (*P* < 0.05) with PNR and NO_3_^–^-N in plow pan soil layer and had significant negative correlation with NH_4_^+^-N.

**FIGURE 6:**
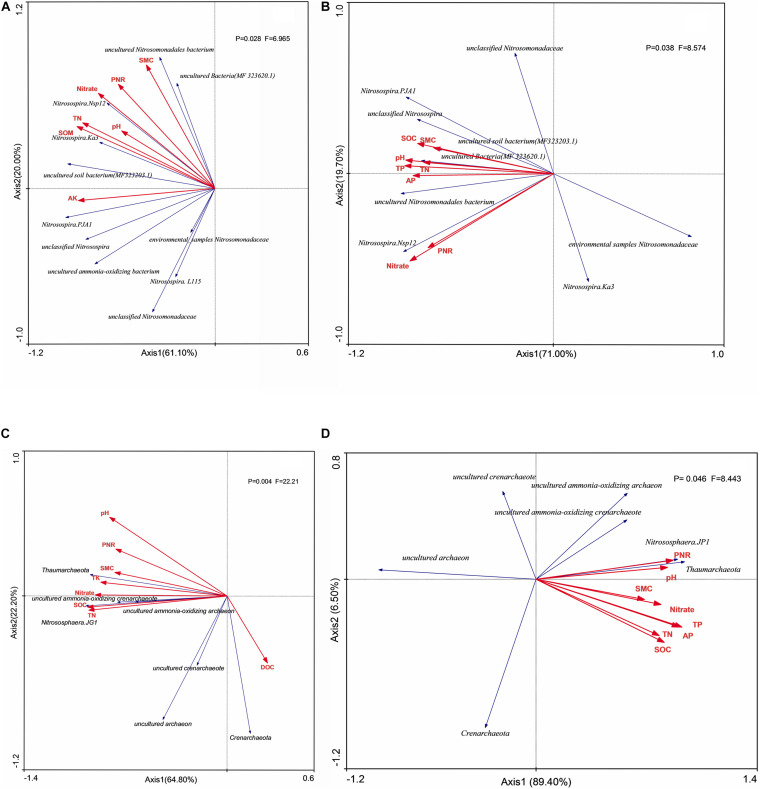
Redundancy analysis (RDA) of AOB taxa on surface arable soil **(A)** and plow pan soil **(B)**, respectively, and of AOA taxa on surface arable soil **(C)** and plow pan soil **(D)**. RDA correlation plot showed variance in taxa of ammonia-oxidation microorganisms explained by soil environmental factors. SOC, soil organic carbon; TN, total nitrogen; TP, total phosphorus; TK, total potassium; AP, available phosphorus; AK, available potassium; NO_3_^–^-N, nitrate nitrogen; PNR, potential nitrite rate; DOC, dissolved organic carbon; SMC, soil moisture carbon.

Shannon index of AOA had no relationship with environmental factors in both two soil layers (Figure 5), whereas the Shannon index of AOB had significant positive correlation (*P* < 0.05) with stocks of SOC, TN, and AK in both two soil layers. Unclassified *Nitrosomonadaceae*, *Nitrosomonadaceae*, and *Nitosospira*.L115 had no significant correlation (*P* > 0.05) with environmental factors, whereas uncultured bacterium MF323203.1, uncultured *Nitrosomonadaceae* bacterium, and *Nitrosospira*.Nsp12 had significant positive correlation (*P* < 0.05) with PNR and stocks of SOC, TN, TP, and NO_3_^–^-N in surface arable soil layer (Figure 6A). Uncultured bacterium MF323203.1 had prominently positive correlation (*P* < 0.05) with TK, AP, and AK, whereas *Nitrosospira*.PJA1 had prominent correlation (*P* < 0.05) with SOC, TK, AP, and AK, and unclassified *Nitrosospira* only had significant correlation (*P* < 0.05) with AK. *Nitrosospira*.Ka3, uncultured bacterium MF323203.1, and environmental samples of *Nitrosomonadaceae* had no correlation (*P* < 0.05) with environmental factors. Uncultured *Nitrosomonadaceae* bacterium and unclassified *Nitrosospira* had significant positive correlation (*P* < 0.05) with PNR and stocks of TP, AP, and NO_3_^–^-N in plow pan soil layer (Figure 6B). Uncultured *Nitrosomonadaceae* bacterium had prominent correlation with soil pH. Uncultured bacterium MF323203.1, uncultured bacterium MF323620.1, and unclassified *Nitrosospira* were significantly correlated with SOC, TP, TK, AP, and AK, whereas *Nitrosospira*.PJA1 had significant correlation with pH, SOC, and TP.

*Crenarchaeota*, unclassified AOA, uncultured *Crenarchaeota*, and uncultured archaea had no significant correlation (*P* < 0.05) with the environmental factors, whereas *Nitrososphaera*.JG1 and *Thaumarchaeota* had significant positive correlation (*P* < 0.05) with PNR, soil pH, and stocks of SOC, TN, TP, TK, and NO_3_^–^-N in surface arable soil layer (Figure 6C). Unclassified AOA, uncultured ammonia-oxidizing *Crenarchaeota*, *Nitrososphaera*.JG1, and *Thaumarchaeota* had significant positive correlation (*P* < 0.05) with PNR and soil pH in plow pan soil layer, whereas *Nitrososphaera*.JG1 and *Thaumarchaeota* had significant positive correlation (*P* < 0.05) with soil pH and stocks of SOC, TN, TP, AP, AK, and NO_3_^–^-N in plow pan soil layer (Figure 6D).

## Discussion

Long-term application of manure with chemical fertilizer could effectively increase soil nutrient content, improve soil physicochemical properties, and increase crop yields, thereby influencing microorganisms (Oehl et al., 2004; Shi et al., 2008; Feitosa de Souza et al., 2016; Lin et al., 2018), whereas long-term application of chemical nitrogen fertilizer alone was likely to decrease soil pH and microbial biomass and damage soil microbial structure (Sun et al., 2017; Huang et al., 2018; Li et al., 2019). Our experiment showed the same results, and the phosphorus fertilizer application could increase organic matters of soil (Table 1). Long-term application of manure with chemical fertilizer effectively slowed down soil acidification and increased SOC, TN, TP, AP, and NO_3_^–^-N in both soil layers, which might be due to the application of manure that could effectively improve physicochemical property of soil.

Our experiment showed that application of organic fertilizer could significantly increase the abundance of *amo*A gene in both soil layers (Figure 1). This result was consistent with results reported as the abundance of AOB in organic fertilizer treatment was higher than that in chemical fertilizer (Li et al., 2017, 2018), which was due to the enrichment of soil nutrients caused the increase of bacterial abundance. Our result has supported the above conjecture, and RDA in abundance of *amo*A gene with environment showed that the abundance of *amo*A gene was significant positively correlated with the stocks of SOC, TN, TP, AP, AK, and NO_3_^–^-N in both layers of soil (Figure 5).

The results of absolute abundance (Figures 1, 2) and relative abundance (Supplementary Figures S1, S2) analysis were different from each other on community structure of ammonia-oxidizing microorganisms. We adopted the combined determination results of absolute abundance with the proportion of relative abundance of each taxon in different treatments for subsequent analysis in order to get the analysis results closer to the real situation.

The application of organic fertilizer, balanced application of chemical fertilizer, and rational application of nitrogen fertilizer could improve the abundance of *arch-amo*A gene in surface arable soil (Figure 2A), whereas the application of organic fertilizer and nitrogen fertilizer could keep the abundance of *arch-amo*A gene constant in plow pan soil layer (Figure 2B). The abundance of *arch-amo*A gene was higher in plow pan soil layer than that in surface arable soil layer. This might be due to the oxygen content in surface arable soil layer being higher than that in plow pan soil layer, and parts of AOA belong to anaerobic heterotrophic bacteria under our experimental condition. Previous studies have confirmed that AOA could adapt to lower oxygen conditions (Liu et al., 2015), and AOB quantities were more easily decreased along with the sediment depth (Wang et al., 2014).

The results of NMDS (Figure 4) showed that the community structure of AOB was divided into two parts in all treatments according to soil layers. This result indicated that the taxa of AOB in different soil layers were significantly different. On the one hand, soil nutrient of plow pan soil layer was significantly lower than that of surface arable soil layer; on the other hand, dissolved oxygen could greatly affect the growth of bacteria (Canziani et al., 2006), whereas AOB were significantly increased in less bioturbated zone (Chen and Gu, 2017). Therefore, community structure of AOB in plow pan soil layer was significantly different from that in surface arable soil layer due to less soil nutrient, oxygen, and disturbance in plow pan soil layer.

The results of NMDS (Figure 4A) showed that the community structure of AOA was divided into two parts in both soil layers due to application of organic fertilizer. This result indicated that AOA had different requirements for nutrient forms. This was consistent with the result reported for readily mineralized organic matter, which was important for AOA (Jiang et al., 2012), and addition of glucose could influence the community of AOA in Beijing Miyun Reservoir (Wang et al., 2015).

PNR was the ability of soil to oxidize ammonium nitrogen to nitrate nitrogen. In our experiment, long-term application of organic fertilizer was more conducive to improving soil PNR; this might be due to that organic fertilizer enhanced the PNR, whereas chemical fertilizer suppressed PNR (Fan et al., 2011). Han et al. (2016) found that PNR was significant positively correlated with abundance of AOB but not AOA. Some studies (Qin et al., 2012; Xu et al., 2012) found that AOA, but not AOB, was significantly correlated with PNR in acidic soils of slope land, and Nguyen et al. (2018) found that PNR was significantly correlated with both AOA and AOB. However, we found that PNR was significantly correlated with the abundance of *amo*A of AOB in surface arable soil layer (Figure 5A) and significantly correlated with the abundance of AOA in plow pan soil layer (Figure 5B). The abundance of uncultured bacterium MF323203.1, uncultured *Nitrosomonadaceae* bacterium, and *Nitrosospira*.Nsp12 had significant correlation with PNR and were mostly influenced by the soil phosphorus (Figures 6A,B), whereas the stock of P was significantly higher in surface arable soil than that in plow pan soil (Table 1). Thus, we hypothesized that this might be related to the beneficial effects of phosphorus on the growth and reproduction of these AOB. The abundance of unclassified AOA, uncultured ammonia-oxidizing *Crenarchaeota*, *Nitrososphaera*.JG1, and *Thaumarchaeota* had significant correlation with PNR and was prominently higher in manure fertilizer treatments than that in chemical fertilizer treatments (Figures 2A,B). NMDS ordinations (Figure 4A) showed that AOA was divided into two parts by organic fertilizer. Thus, we hypothesized that abundance of these AOA could be increased by complex changes in the soil physical and chemical properties, which were due to organic fertilizer. These results might be due to that uncultured bacterium MF323203.1, uncultured *Nitrosomonadaceae* bacterium, *Nitrosospira*.Nsp12, *Nitrososphaera*.JG1, and *Thaumarchaeota* played a dominant role in PNR (Figure 6). However, the changes of abundance of uncultured bacterium MF323203.1, uncultured *Nitrosomonadaceae* bacterium, and *Nitrosospira*.Nsp12 that were positively correlated with PNR were consistent with the total abundance of AOB. The changes of abundance of *Nitrososphaera*.JG1 and *Thaumarchaeota* that had positive correlation with PNR were different from the total abundance of AOA in surface arable soil layer. The changes of abundance of unclassified AOA, uncultured ammonia-oxidizing *Crenarchaeota*, *Nitrososphaera*.JG1, and *Thaumarchaeota* were positively correlated with PNR and consistent with the total abundance of AOA. The changes of abundance of uncultured *Nitrosomonadaceae* bacterium and *Nitrosospira*.NSP12 were positively correlated with PNR and different from the total abundance of AOB in plow pan soil layer. PNR was correlated with unclassified AOA, uncultured ammonia-oxidizing *Crenarchaeota*, *Nitrososphaera*.JG1, *Thaumarchaeota* (AOA) and uncultured bacterium MF323203.1, uncultured *Nitrosomonadaceae* bacterium, and *Nitrosospira*.Nsp12 (AOB). PNR was also positively related to the abundance of AOB in surface arable layer and AOA in plow pan layer macroscopically. The essence was that these taxa affected the ammonia oxidation under long-term fertilization in brown soil. It might be a good explanation for some studies suggesting that AOA or AOB played a dominant role in various environments.

## Conclusion

In conclusion, long-term fertilization changed soil PNR by influencing specific ammonia-oxidizing microorganisms, which are influenced by soil physiochemical properties in the brown soil. Only specific and rare AOA and AOB instead of the whole AOA or AOB community played a dominant role in nitrification in brown soil under long-term fertilization.

## Data Availability Statement

The datasets presented in this study can be found in online repositories. The names of the repository/repositories and accession number(s) can be found in the article/Supplementary Material.

## Author Contributions

FC, XH, and PL conceived, designed the study, and wrote the manuscript. JY, MI, SZ, NA, and JD collected and analyzed the data. All authors read and approved the final manuscript.

## Conflict of Interest

The authors declare that the research was conducted in the absence of any commercial or financial relationships that could be construed as a potential conflict of interest.
