# Supplementary material for: Effect of Long-Term Fertilization on Ammonia-Oxidizing Microorganisms and Nitrification in Brown Soil of Northeast China
Source: Front Microbiol. 2021 Feb 4;11:622454. doi: 10.3389/fmicb.2020.622454 (PMC7890093; doi:10.3389/fmicb.2020.622454)
Supplement: Supplementary file 1 [file Data_Sheet_1.docx]

**Fig.s1** The community structure under relative abundance of ammonia-oxidizing bacteria in different treatments in surface arable soil (a) and plough pan soil (b), respectively.





**Fig.s2** The community structure under relative abundance of ammonia-oxidizing archaea in different treatments in surface arable soil (a) and plough pan soil (b), respectively.
