# Supplementary material for: Effect of Long-Term Fertilization on Ammonia-Oxidizing Microorganisms and Nitrification in Brown Soil of Northeast China
Source: Front Microbiol. 2021 Feb 4;11:622454. doi: 10.3389/fmicb.2020.622454 (PMC7890093; doi:10.3389/fmicb.2020.622454)
Supplement: Supplementary file 2 [file Data_Sheet_2.docx]

**Table S1** Application rates of fertilizer in maize and soybean of the treatments

| Treatments | Chemical fertilization rates (kg/hm^2^) | | | Pig manure(t•ha^-1^) |
| --- | --- | --- | --- | --- |
|  | N | P_2_O_5_ | K_2_O_2_ |  |
| CK | 0/0 | 0/0 | 0/0 | 0/0 |
| N | 120/30 | 0/0 | 0/0 | 0/0 |
| N2 | 180/60 | 0/0 | 0/0 | 0/0 |
| NP | 120/30 | 60/90 | 0/0 | 0/0 |
| NPK | 120/30 | 60/90 | 60/90 | 0/0 |
| M | 0/0 | 0/0 | 0/0 | 27/0 |
| MN | 120/30 | 0/0 | 0/0 | 27/0 |
| MN2 | 180/60 | 0/0 | 0/0 | 27/0 |
| MNPK | 120/30 | 60/90 | 60/90 | 27/0 |

Note：The long-term fertilization started since 1979, the rotation system was maize-maize-soybean, the rotation was repeated every three years. Abbreviations: CK, no fertilizer, N, mineral nitrogen fertilizer; N_2_, high mineral nitrogen fertilizer; NP , mineral nitrogen and phosphate fertilizer; NPK, mineral nitrogen, phosphate and potassium fertilizer; M, pig manure; MN, pig manure and mineral nitrogen fertilizer; MN_2_, pig manure and high mineral nitrogen fertilizer; MNPK, pig manure, mineral phosphate, potassium fertilizer and mineral nitrogen fertilizer

**Table S2** The pairs of primer of *amo*A gene of AOA and AOB

| Primers | Sequence | | References |
| --- | --- | --- | --- |
| Arch-*amo*AF | 5'-STAATGGTCTGGCTTAGACG-3' | Francis et al.(2005) | |
| Arch-*amo*AR | 5'-GCGGCCATCCATCTGTATGT-3' |  |  |
| amoA-1F | 5'-GGGGTTTCTACTGGTGGT-3' | Rotthauwe et al.(1997) | |
| amoA-2R | 5'-CCCCTCKGSAAAGCCTTCTTC-3' |  |  |

**Table S3** The differences of AOB abundance in species level between fertilization treatments

|  | Soil layers | CK | N | N_2_ | NP | NPK | M | MN | MN_2_ | MNPK |
| --- | --- | --- | --- | --- | --- | --- | --- | --- | --- | --- |
| uncultured soil bacterium (MF323203.1) | 0-20cm | d | d | d | d | c | c | a | b | a |
|  | 20-40cm | cd | b | a | cd | b | de | e | c | f |
| uncultured bacteria  (MF 323620.1) | 0-20cm | a | b | b | b | b | b | b | b | b |
|  | 20-40cm | c | c | d | ab | c | d | a | d | b |
| unclassified Nitrosomonadaceae | 0-20cm | c | b | de | a | a | e | cde | cd | cde |
|  | 20-40cm | d | d | d | d | b | a | c | b | d |
| environmental_samples Nitrosomonadaceae | 0-20cm | b | b | a | b | b | b | b | b | b |
|  | 20-40cm | c | e | e | e | e | d | b | b | a |
| Nitrosospira.Ka3 | 0-20cm | bc | a | bc | ab | c | bc | abc | ab | bc |
|  | 20-40cm | a | e | f | b | bc | f | f | de | cd |
| uncultured Nitrosomonadales bacterium | 0-20cm | b | bc | c | c | c | a | bc | bc | bc |
|  | 20-40cm | bc | e | e | cd | d | cd | b | b | a |
| uncultured ammonia-oxidizing bacterium | 0-20cm | b | b | b | ab | a | b | a | a | a |
| Nitrosospira. L115 | 0-20cm | b | a | ab | ab | ab | b | b | b | b |
| unclassified Nitrosospira | 0-20cm | a | a | a | a | a | a | a | a | a |
|  | 20-40cm | a | b | b | c | c | c | c | c | c |
| Nitrosospira.Nsp12 | 0-20cm | b | b | b | b | b | a | b | b | b |
|  | 20-40cm | d | d | d | c | d | d | b | c | a |
| Nitrosospira.PJA1 | 0-20cm | e | e | e | d | c | de | a | ab | bc |
|  | 20-40cm | d | d | d | d | d | a | c | b | d |

Note: Different letters in the same row represent significantly differences among fertilization treatments (LSD method, and p<0.05).

**Table S4** The differences of AOA abundance in species level between fertilization treatments

|  | Soil layers | CK | N | N_2_ | NP | NPK | M | MN | MN_2_ | MNPK |
| --- | --- | --- | --- | --- | --- | --- | --- | --- | --- | --- |
| Crenarchaeota | 0-20cm | de | b | a | c | d | e | d | c | c |
|  | 20-40cm | d | e | g | c | a | b | ef | f | f |
| unclultured ammonia-oxidizing archaeon | 0-20cm | b | c | e | b | a | b | c | c | d |
|  | 20-40cm | f | b | a | c | d | f | e | b | c |
| uncultured ammonia-oxidizing crenarchaeote | 0-20cm | a | e | f | cd | bc | bc | b | cd | de |
|  | 20-40cm | a | de | e | d | d | c | b | d | d |
| Nitrososphaera.JG1 | 0-20cm | de | e | e | c | cd | a | a | a | b |
|  | 20-40cm | ef | f | f | f | e | a | b | d | c |
| uncultured crenarchaeote | 0-20cm | a | b | de | bc | b | e | c | d | de |
|  | 20-40cm | a | bc | de | bc | b | de | cd | e | de |
| Thaumarchaeota | 0-20cm | d | d | d | d | d | a | b | c | b |
|  | 20-40cm | d | e | e | d | d | c | a | c | b |
| uncultured archaeon | 0-20cm | d | bc | d | cd | cd | d | b | a | bcd |
|  | 20-40cm | c | a | b | c | c | d | d | d | d |

Note: Different letters in the same row represent significantly differences among fertilization treatments (LSD method, and p<0.05).
